# A Few Words of Explanation from Prof. James P. White, M. D.

**Published:** 1878-02

**Authors:** James P. White

**Affiliations:** 674 Main St., Buffalo


MESSRS EDITORS:*—Permit me space in your valuable JOURNAL for a few words of explanation. If there is any one thing, about which I have been, through life, over cautious it is in allowing my name to be used in commendation of any quack remedy or of any syrup combination or mineral water or of any place as a sanitarium, etc., etc. So much do all good remedies depend, for their usefulness, upon their adaptation to overcome the symptoms present in the individual case, and so liable is every recommendation to be perverted or misconstrued, that my rule is invariably to refuse the use of my name. Forty years ago I refused to subscribe to a paper recommending Bristol's Sarsaparilla, which certificate had already received the signatures of all my fellow practitioners, and gave great offense to the parties interested in its sale, and this course has been ever since adhered to.

Notwithstanding all my caution I am caught at last. Some one has kindly sent me a "poster" in which I am with Prof's. Thomas, Byford, Brickell, and others given as authority for the usefulness of a remedy called the "Boudoir." This wonderful remedy is made and advertised for sale by Dr. Cronyn, of Dunkirk, N. Y. This gentleman who is a graduate of the Buffalo Medical College, and was in good standing, so far as I know, as a practitioner, addressed me a note some time last year, enclosing a recipe for a preparation containing some form of Iron and Bark with perhaps other ingredients, asking my opinion of its utility as a tonic in treating, as I understood, some case of anæmia, which he had then under his care. Without a suspicion that I was being entrapped by an old student into recommending a panacea I unhesitatingly answered that it was a good combination or something to that end; perhaps I said it was "admirable." It is not unlikely that Prof's. Thomas, Byford and the other gentlemen whose names are attached to the recommendations of the "Boudoir" have been similarly caught. It is certainly a very ingenious method of proceeding, whatever one may think of its fairness. With all my caution I have been very shrewedly "victimized." Truly Yours, JAMES P. WHITE.

674 Main St., Buffalo.

* This timely explanation is received in season to answer *our* inquiries for the medicinal properties of "Boudoir."—*Ed.*

